# Correlation of endoscopic third ventriculostomy with postoperative body temperature elevation: a single-center retrospective comparative study

**DOI:** 10.1007/s10143-025-03190-0

**Published:** 2025-01-10

**Authors:** Mohammed Issa, Clara Dannehl, Angelika Seitz, Pavlina Lenga, Steffen Syrbe, Sandro M. Krieg, Ahmed El Damaty

**Affiliations:** 1 Faculty of Medicine, Heidelberg University, Heidelberg, Germany; 2 Department of Neurosurgery, Heidelberg University Hospital, Heidelberg, Germany; 3 Center for Child and Adolescent Medicine, Clinic I, Division of Pediatric Epileptology, University Hospital Heidelberg, Heidelberg, Germany; 4 Deptartment of Neuroradiology, Heidelberg University Hospital, Heidelberg, Germany

## Abstract

Postoperative fever following neuroendoscopic procedures has been well-documented, yet specific differentiation based on the nature and site of the procedure remains lacking. Given the anatomical involvement of the hypothalamus in temperature regulation, we propose that endoscopic third ventriculostomy (ETV) may have a distinct impact on postoperative fever. This study aims to investigate this phenomenon. This retrospective comparative analysis includes all patients who underwent neuroendoscopic procedures between January 2017 and September 2023. Patients were divided into ETV and non-ETV groups, and comparisons were made regarding postoperative body temperature during the initial 7 days after surgery. Comprehensive data were collected on case numbers, surgical duration, symptoms, treatments, and outcomes. Body temperature was measured postoperatively in the morning and evening for 7 days, with elevated temperature categorized as sub-fever (37.5 to 38.2 °C) and fever (≥ 38.3 °C). 207 patients underwent neuroendoscopic procedures in our neurosurgical centers (median age19.1 ± 21.7 years, 50.7% male), primarily for aqueduct stenosis (25.6%) and intra- and periventricular tumors (25.1%). Among them, 104 (50.2%) patients underwent ETV, while 103 (49.8%) underwent other neuroendoscopic procedures (43.7% intracranial cysts fenestrations, 39.8% placement of intraventricular catheters, 3.9% intraventricular lavage, 4.9% septostomy, and 5.8% tumor biopsy). All postoperative infections were excluded. No significant differences were observed in preoperative symptoms, laboratory findings, or postoperative antibiotic usage between the two groups. The ETV group exhibited significantly more postoperative fever (37.5% vs. 19.4%, *p* = 0.005), particularly from the first night to the third night after the operation. This study substantiates the hypothesis that manipulation of the floor of third ventricle through endoscopic ventriculostomy may contribute to postoperative fever, rather than the neuroendoscopic procedure. Elevated temperatures were observable from the first night post-surgery and typically normalized by third day without necessitating specific treatment. Further prospective studies are warranted to elucidate the precise mechanisms underlying intraoperative manipulation.

## Introduction

Postoperative fever is a common occurrence in children following surgery, affecting between 33% and 50% of patients [2, 16]. Typically, the fever manifests within 12 to 72 h after the procedure and is influenced by factors such as the duration and type of surgery, anesthesia method, intraoperative blood transfusions, and the use of preoperative antibiotics [11, 22]. The primary goal of evaluating postoperative fever is to distinguish between fever caused by an infection and that due to non-infectious causes [15].

The exact pathophysiology behind non-infectious postoperative fever is not entirely understood, but it is believed to result from the body’s response to the release of stress hormones and cytokines [14, 17]. Notably, an increase in body temperature has been particularly observed in pediatric neurosurgical patients, especially following endoscopic procedures in the intracranial or intraventricular space. However, there is limited differentiation based on the specific nature and location of the procedure [3, 9, 18].

**Fig. 1:**
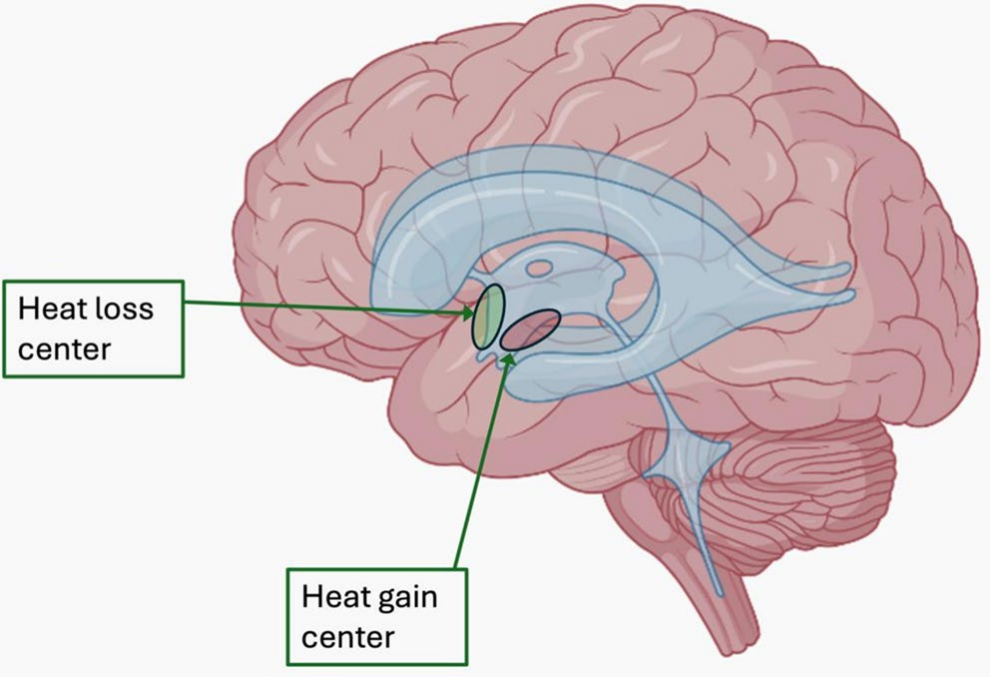
Illustration of the heat loss center in the anterior hypothalamus and the heat gain center in the posterior hypothalamus

Although infections are often not the cause of these fevers, antibiotics are frequently prescribed, leading to prolonged hospital stays [9]. The center responsible for development of postoperative fever, particularly after neuroendoscopic procedures, is the hypothalamus, which regulates body temperature [3, 9]. Current studies focus on the role of the anterior preoptic and dorsomedial nuclei in this regulation but largely overlook the contribution of the inferior hypothalamus, particularly the floor of the third ventricle [9, 12, 23]. Studies revealed that heat loss center is located in the anterior hypothalamus while heat gain center is located in the posterior hypothalamus [6], see Fig. 1. Normal body temperature is regulated by the thermoregulatory center in the anterior hypothalamus. Prostaglandin E2 (PGE2) is believed to be the primary mediator of the febrile response. Increased intracellular levels of PGE2 within the hypothalamus act as a trigger, raising the temperature set point. This, in turn, activates neurons in the vasomotor center, initiating vasoconstriction and reducing the firing rate of warm-sensitive neurons, thereby promoting heat production in peripheral tissues. Once the hyperthermia stabilizes, any fluctuations in core body temperature will activate thermoregulatory mechanisms, similar to those at normal body temperature, to maintain the elevated set point [19].

Given the role of hypothalamus in temperature regulation, we propose that endoscopic third ventriculostomy (ETV) may have a distinct influence on postoperative fever. This study aims to clarify the possible involvement of hypothalamic nuclei in temperature elevation following these procedures and further investigate this phenomenon.

## Methods

### Materials and methods

This single-center retrospective study included patients who underwent neuroendoscopic procedures for intra- and periventricular pathologies between January 2017 and December 2023. The study was conducted in accordance with the principles of the Declaration of Helsinki and received approval from the University Ethics Committee (approval number: S-084/2022). Informed consent was obtained from all patients or their legal guardians. During endoscopic procedures, we used warm lactate-free Ringer solution warmed to 37 °C. The surgical cases were divided into two groups. The study group comprised patients who underwent endoscopic third ventriculostomy (ETV). Some patients in this group also underwent additional neuroendoscopic procedures, for example additional septostomy or tumor biopsy. We included these patients in the ETV group as it supports our hypothesis due to primarily involving manipulation of the hypothalamus at the floor of the third ventricle done through ETV procedure. The stoma in the third ventricular floor was created uniformly across all ETV patients using Decq forceps for perforation, followed by dilation with a Fogarty balloon. Electrical coagulation was strictly avoided. The control group consisted of patients who underwent neuroendoscopic procedures without ETV. These procedures included endoscope-assisted ventricular catheter placement, cyst fenestration, septostomy, tumor biopsy, neuroendoscopic lavage of ventricular system, and endoscopic tumor resection.

Postoperatively, body temperature was measured through the ear (auricular) both in the morning and evening whenever fever occurs and persists up to seven days. Patients were discharged when they remained 24 h fever-free. Fever was categorized as sub-fever (37.5 °C to 38.2 °C) and fever (≥ 38.3 °C) [10]. All patients received standard perioperative antibiotics (2nd generation Cephalosporins) during the first two postoperative days. All patients were treated using standardized medical protocols for pain management, including Metamizole (Novalgina^®^) and NSAIDs, as well as for controlling postoperative temperature elevations. CRP levels were measured as a baseline in all patients with hyperthermia and reassessed after two days for monitoring. Patients with proven infection, clinically and through laboratory investigations, within the first seven days or with incomplete body temperature documentation were excluded from the study.

All patients were routinely discharged from the hospital two to three days after undergoing neuroendoscopy, provided no complications arose. In cases where fever developed, discharge was delayed until the patient had been free of fever for at least 24 h.

A comprehensive analysis was carried out, examining variables such as patient age, gender, follow-up duration, pathology, operative technique, cerebrospinal fluid (CSF) laboratory results, and preoperative symptoms. Both surgery-related and non-surgery-related adverse events were recorded, along with mortality rates.

Standard clinical and radiological follow-up was conducted before discharge, at three months post-surgery, and during the final follow-up. Success of the neuroendoscopic procedures was evaluated using standard brain Magnetic Resonance Imaging (MRI).

A subanalysis of pediatric and adult patients was conducted, with a detailed evaluation of postoperative fever patterns, pathologies, surgical techniques, and complication rates. This subanalysis involved a thorough examination of the specific pathologies within each age group, the surgical approaches used, and the varying complication rates in the pediatric population.

### Statistical analysis

Normal distribution was assessed using the Shapiro–Wilk test. Continuous variables were presented as mean ± standard deviation, and categorical variables as frequencies and percentages. Intergroup comparisons for continuous variables were performed using the t-test, while the Mann–Whitney U test and Fisher’s exact test were used for categorical variables. Statistical significance was set at *p* < 0.05. All analyses were conducted using SPSS version 29 (IBM Corp, Armonk, NY, USA).

## Results

### Inclusion of patients in the study

**Fig. 2:**
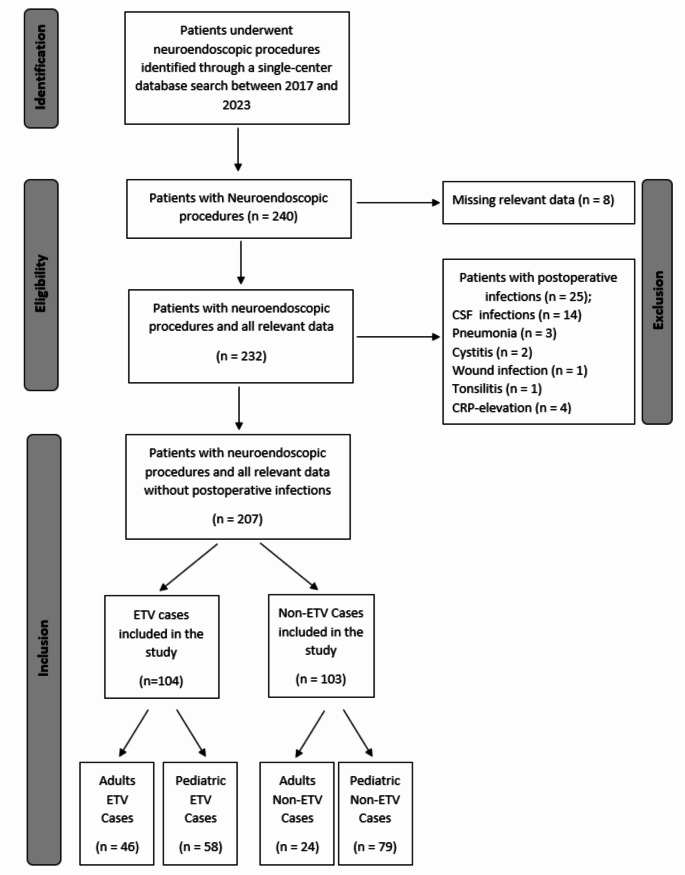
Flow diagram illustrating the process of inclusion and exclusion of the study cohort

The study included 240 patients who underwent neuroendoscopic procedures between 2017 and 2023. After excluding 8 cases with missing data, 25 patients with postoperative infections (e.g., CSF infections, pneumonia, cystitis) during the first seven postoperative days were also excluded. This left 207 patients with complete data, including 104 (50.2%) who underwent endoscopic third ventriculostomy (ETV) and 103 (49.8%) who underwent other neuroendoscopic procedures. The cohort was further divided into pediatric and adult groups, with 58 pediatric and 46 adult ETV cases, and 79 pediatric and 24 adult non-ETV cases. Figure 2, presented as a flowchart, outlines the steps for inclusion and exclusion in the study.

### Patients’ characteristics and intergroups’ comparison, ETV vs. non-ETV

The study included a total of 207 patients, with 104 (50.2%) undergoing endoscopic third ventriculostomy (ETV) and 103 (48.8%) undergoing other neuroendoscopic procedures without ETV. The gender distribution showed 105 males (50.7%) and 102 females (49.3%), with no significant difference between the ETV and non-ETV groups (*p* = 0.127), the mean age of patients was 19.1 ± 21.7 years, with those in the ETV group being significantly older (23.1 ± 23.4 years) compared to the non-ETV group (15.0 ± 19.1 years, *p* = 0.007). Pediatric patients comprised 66.2% of the total cohort, with a higher proportion in the non-ETV group (76.7%) compared to the ETV group (55.8%). The mean follow-up duration was longer in the ETV group (34.9 ± 23.3 months) than in the non-ETV group (27.9 ± 21.8 months, *p* = 0.026), Table 1.

Surgical duration was significantly shorter in the ETV group (68.9 ± 47.9 min) compared to the non-ETV group (86.8 ± 42.5 min, *p* = 0.005). Patients in the ETV group had a shorter ICU stay (0.7 ± 1.6 days) compared to the non-ETV group (2.0 ± 4.4 days, *p* = 0.005), the most common etiologies were arachnoid and ependymal cysts (26.1%), intraventricular tumors (25.1%), idiopathic aqueductal stenosis (25.6%), and posthemorrhagic hydrocephalus (17.9%). ETV was more frequently performed in patients with idiopathic aqueductal stenosis (42.3%) and intraventricular tumors (32.7%, *p* < 0.001), Table 1.

Postoperative clinical improvement was seen in 85.5% of patients, with no significant difference between the ETV (87.5%) and non-ETV (83.4%) groups (*p* = 0.437). Radiological improvement was observed in 83.6% of patients, again with no significant difference between the groups (*p* = 0.459). Postoperative hygromas occurred in 6.8% of cases, with no significant difference between the groups (*p* = 0.284). Laboratory analysis of cerebrospinal fluid (CSF) showed no significant differences in protein, lactate, or leukocyte content between groups, but glucose levels were higher in the ETV group (*p* = 0.001), Table 1.

**Table 1:** **Patient**’s characteristics und comparison between **ETV**- and **Non-ETV**

Variable Cases	Cases 207 (100)	ETV 104 (50.2)	Non-ETV 103 (49.8)	*p*-value
**Sex**				
Male	105 (50.7)	47 (45.2)	58 (56.3)	0.127
Female	102 (49.3)	57 (54.8)	45 (43.7)	
Age* **in years**	19.1 ± 21.7	23.1 ± 23.4	15.0 ± 19.1	**0.007**
**Pediatric patients**	137 (66.2)	58 (55.8)	79 (76.7)	**0.002**
Surgery duration* in minutes	77.8 ± 46.1	68.9 ± 47.9	86.8 ± 42.5	**0.005**
ICU stay* in days	1.3 ± 3.4	0.7 ± 1.6	2.0 ± 4.4	**0.005**
**Postoperative body temperature elevation > 37.5 °C**	126 (60.9)	74 (71.2)	52 (50.5)	**0.003**
**Postoperative Subfebrile 37.5–38.29 °C**	67 (32.4)	35 (33.7)	32 (31.1)	0.767
**Postoperative Fever > 38.29 °C**	59 (28.5)	39 (37.5)	20 (19.4)	**0.005**
**Neuroendoscopic procedures**, ***n* = 207**:				
Endoscopic third ventriculostomy (ETV)	104 (50.2)	104 (100.0)	0 (0.0)	**< 0.001**
Ventricular catheter placement	41 (19.8)	0 (0.0)	41 (39.8)	
Cysts fenestration	45 (21.7)	0 (0.0)	45 (43.7)	
Septostomy	5 (2.4)	0 (0.0)	5 (4.9)	
Tumor biopsy	6 (2.9)	0 (0.0)	6 (5.8)	
Ventricular lavage	4 (1.9)	0 (0.0)	4 (3.9)	
Endoscopic tumor resection	2 (1.0)	0 (0.0)	2 (1.9)	
**Intraoperative CSF examination**,** content in median**,** IQR (Reference)**				
Protein (< 0.4 g/l)	0.14, 0.08	0.25 ± 0.4	0.31 ± 0.42	0.125
Glucose (49–75 mg/dl)	55.0, 47.0	59.0 ± 12.7	49.2 ± 17.4	0.001
Lactate (1.1–1.8 mmol/l)	1.26, 1.09	1.3 ± 0.44	2.6 ± 8.2	0.234
Leukocytes (< 5 /ul)	1.0, 1.0	5.02 ± 13.4	8.7 ± 30.2	0.386

(%) Data in parenthesis are percentages

*Data are given as mean ± standard deviation

### Comparison of body temperature

Postoperative body temperature elevation (above 37.5 °C) occurred more frequently in the ETV group (71.2%) compared to the non-ETV group (50.5%, *p* = 0.003). Fever (≥ 38.3 °C) was more common in the ETV group (37.5%) compared to the non-ETV group (19.4%, *p* = 0.005), though the rates of subfebrile temperatures (37.5–38.29 °C) were not significantly different between the groups (*p* = 0.767), Table 1.

**Fig. 3:**
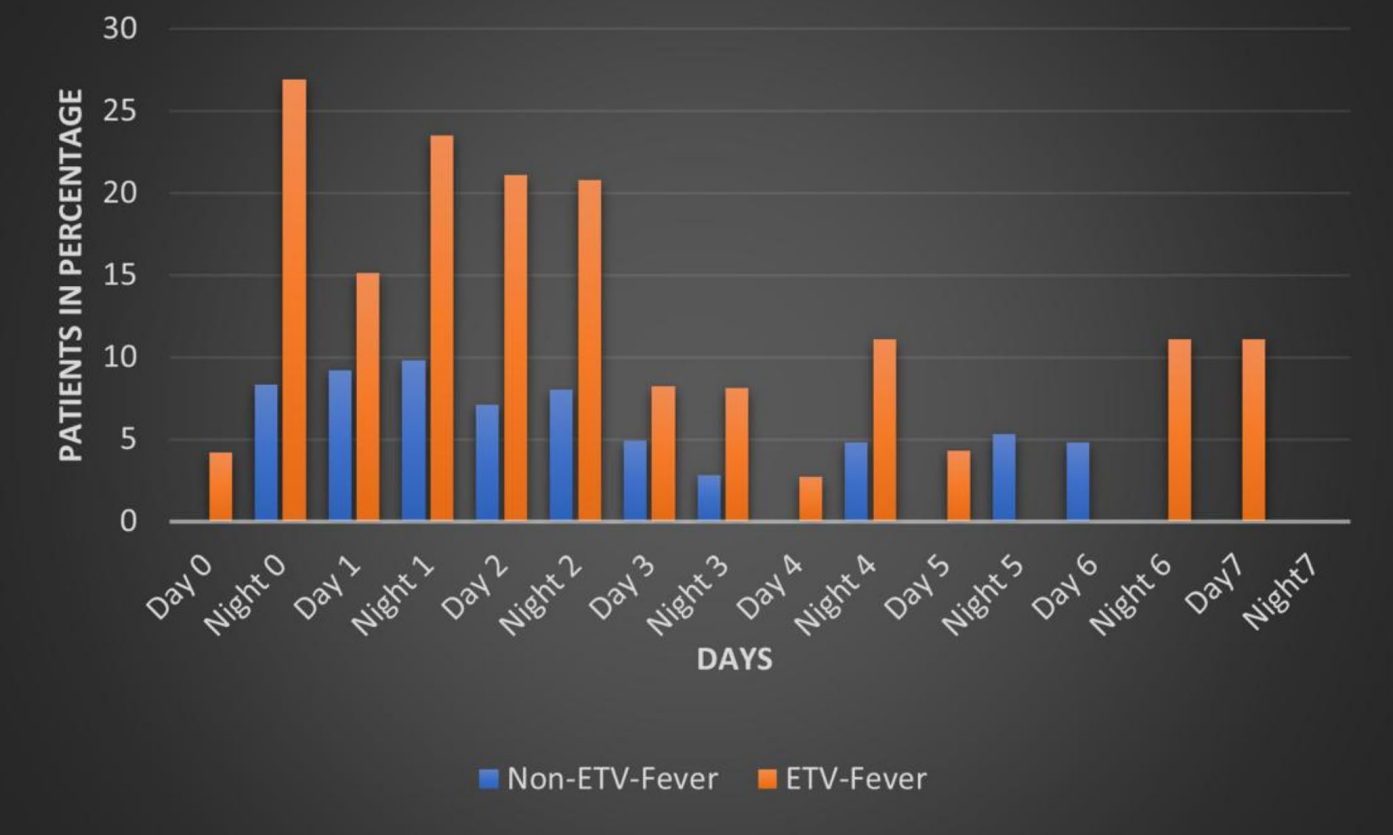
Comparison of fever incidence between the ETV and non-ETV groups over a 7-day postoperative period

Figure 3 presents a comparison of fever incidence between the ETV and non-ETV groups over a 7-day postoperative period, with body temperatures recorded during day and night. In the non-ETV group, fever was rare, starting in 8.3% on Night 0, peaking in 9.8% on Day 1, and then declining gradually to 0% by Day 7. In contrast, the ETV group had a much higher and more variable incidence of fever, beginning in 4.2% on Day 0, peaking in 26.9% on Night 0, and fluctuating throughout the week. Fever incidence in the ETV group also experienced notable increases on Days 4 and 7, with 11.1% recorded during those nights.

### Sub-analysis in pediatric patients with intragroup compression between ETV and non-ETV

The study included 137 pediatric patients, with 58 (42.3%) undergoing endoscopic third ventriculostomy (ETV) and 79 (57.7%) undergoing non-ETV neuroendoscopic procedures. Postoperative body temperature elevation (above 37.5 °C) was significantly more frequent in the ETV group, occurring in 87.9% of patients, compared to 51.9% in the non-ETV group (*p* < 0.001). Fever (above 38.3 °C) was also notably higher in the ETV group, affecting 53.5% of patients, whereas only 21.5% of the non-ETV patients experienced fever (*p* < 0.001). However, the rate of subfebrile temperature (37.5–38.2 °C) was not significantly different between the groups (*p* = 0.712).

The study also found that the ETV group had a shorter ICU stay (0.7 ± 1.4 days) compared to the non-ETV group (2.2 ± 4.7 days, *p* = 0.007), while the surgery duration, age, and follow-up periods showed no significant differences. Surgical complications were more common in the ETV group (8.6% vs. 1.3%), although this difference was not statistically significant (*p* = 0.083). for more details see Table 2.

**Table 2:** Pediatric **Patient**’s characteristics und comparison between **ETV**- and **Non-ETV**

Variable Cases	Cases 137 (100)	ETV 58 (42.3)	Non-ETV 79 (57.7)	*p*-value
**Sex**				
Male	69 (50.4)	27 (46.6)	42 (53.2)	0.491
Female	68 (49.6)	31 (53.4)	37 (46.8)	
Age* **in years**	5.5 ± 5.2	5.2 ± 5.2	5.8 ± 5.2	0.510
Surgery duration* in minutes	84.6 ± 51.3	80.3 ± 59.3	87.8 ± 44.7	0.422
ICU stay* in days	1.6 ± 3.7	0.7 ± 1.4	2.2 ± 4.7	**0.007**
**Postoperative body temperature elevation > 37.5 °C**	92 (67.2)	51 (87.9)	41 (51.9)	**< 0.001**
**Postoperative Subfebrile 37.5–38.2 °C**	44 (32.1)	20 (34.5)	24 (30.4)	0.712
**Postoperative Fever > 38.2 °C**	48 (35.0)	31 (53.5)	17 (21.5)	**< 0.001**
**Neuroendoscopic procedures**, ***n* = 207**:				
Endoscopic third ventriculostomy (ETV)	58 (42.3)	58 (100.0)	0 (0.0)	**< 0.001**
Ventricular catheter placement	37 (27.0)	0 (0.0)	37 (46.9)	
Cysts fenestration	30 (21.9)	0 (0.0)	30 (38.0)	
Septostomy	5 (3.6)	0 (0.0)	5 (6.3)	
Tumor biopsy	2 (1.5)	0 (0.0)	2 (2.5)	
Ventricular lavage	4 (2.9)	0 (0.0)	4 (5.1)	
Endoscopic tumor resection	1 (0.7)	0 (0.0)	1 (1.3)	
**Intraoperative CSF examination**,** content in median**,** IQR (Reference)**				
Protein (< 0.4 g/l)	0.14, 0.20	0.24 ± 0.4	1.7 ± 7.3	0.134
Glucose (49–75 mg/dl)	54.0, 1.0	55.9 ± 10.2	50.0 ± 15.4	0.026
Lactate (1.1–1.8 mmol/l)	1.23, 0.76	1.2 ± 0.2	1.5 ± 0.6	0.003
Leukocytes (< 5 /ul)	2.0, 1.0	3.7 ± 4.5	9.5 ± 32.1	0.195

(%) Data in parenthesis are percentages

*Data are given as mean ± standard deviation

**Fig. 4:**
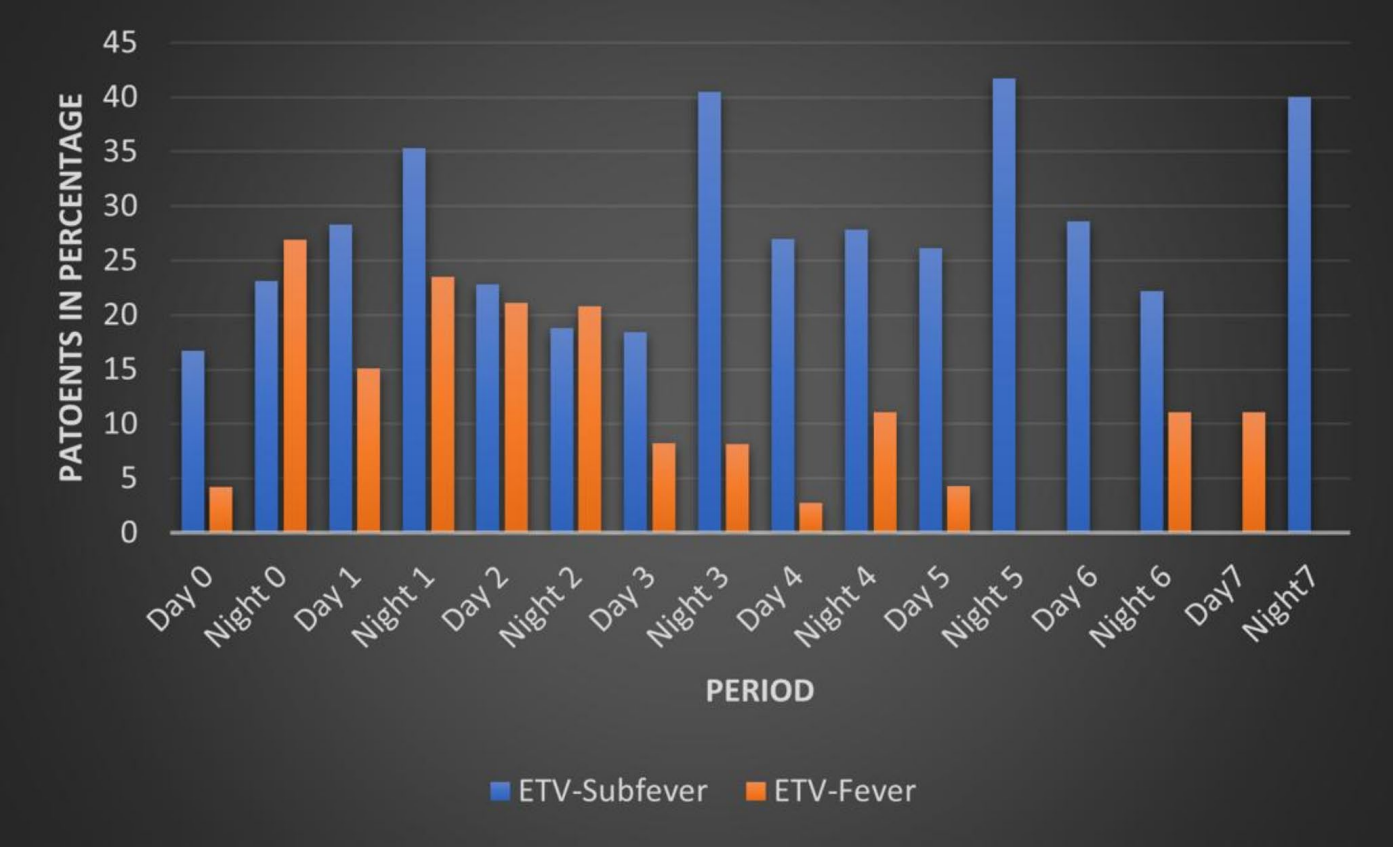
Postoperative Body temperature elevation in Children with ETV

Figure 4 illustrates the incidence of subfever and fever in the ETV group over a 7-day postoperative period, with body temperatures measured both during the day and night. Subfever was consistently present, beginning in 15% on Day 0 and persisting throughout the 7 days, with peaks around 40% on Nights 3, 5, and 7. In contrast, fever showed a more variable pattern, peaking in 26.9% on Night 0 and fluctuating throughout the week before subsiding after Day 4.

### Sub-analysis in adult patients with intragroup compression between ETV and non-ETV

Table 3 summarizes the characteristics and outcomes of 70 adults’ cases, with 46 (65.7%) undergoing ETV and 24 (34.3%) non-ETV procedures. The groups were similar in age (*p* = 0.915) and sex distribution (*p* = 0.081). ETV patients had a significantly longer follow-up (mean 31.9 vs. 19.1 months, *p* = 0.015) and shorter surgery duration (54.5 vs. 83.7 min, *p* = 0.001), while ICU stays showed no significant difference (*p* = 0.508).

Postoperative temperature elevations were recorded in both groups, with body temperatures > 37.5 °C observed in 51.4% of cases, with 50.0% in the ETV group and 45.8% in the non-ETV group (*p* = 0.804). Specifically, subfebrile temperatures were noted in 32.9% (32.6% in the ETV and 33.3% in the non-ETV group, *p* = 0.737), and postoperative Fever (> 38.2 °C) was observed in 15.7% of cases, with a slightly higher occurrence in the ETV group (17.4%) compared to the non-ETV group (12.5%) (*p* = 0.737).

Adverse events remained low, with surgical adverse events in 4.3% of cases (*p* = 1.0) and non-surgical adverse events also in 4.3% (*p* = 0.269). Clinical improvement was observed in 82.9% of cases and radiological improvement in 85.5%, with no significant difference between ETV and non-ETV groups.

**Table 3:** Adult **Patient**’s characteristics und comparison between **ETV**- and **Non-ETV**

Variable Cases	Cases 70 (100)	ETV 46 (65.7)	Non-ETV 24 (34.3)	*p*-value
Sex				
Male	36 (51.4)	20 (43.5)	16 (66.7)	0.081
Female	34 (48.6)	26 (56.5)	8 (33.3)	
Age* **in years**	45.6 ± 16.5	45.8 ± 16.8	45.3 ± 16.3	0.915
Surgery duration* in minutes	64.5 ± 29.5	54.5 ± 20.3	83.7 ± 34.9	**0.001**
ICU stay* in days	1.6 ± 3.7	0.6 ± 1.8	1.1 ± 3.4	0.508
**Postoperative body temperature elevation > 37.5 °C**	36 (51.4)	23 (50.0)	11 (45.8)	0.804
**Postoperative Subfebrile 37.5–38.2 °C**	23 (32.9)	15 (32.6)	8 (33.3)	0.737
**Postoperative Fever > 38.2 °C**	11 (15.7)	8 (17.4)	3 (12.5)	0.737
**Neuroendoscopic procedures**, ***n* = 70**:				
Endoscopic third ventriculostomy (ETV)	46 (65.7)	58 (100.0)	0 (0.0)	**< 0.001**
Ventricular catheter placement	4 (5.7)	0 (0.0)	4 (16.7)	
Cysts fenestration	15 (21.4)	0 (0.0)	15 (62.5)	
Tumor biopsy	4 (5.7)	0 (0.0)	4 (16.7)	
Endoscopic tumor resection	1 (1.4)	0 (0.0)	1 (4.2)	

(%) Data in parenthesis are percentages

*Data are given as mean ± standard deviation

### Occurrence of fever in relation to age and type of endoscopic procedure

Finally, upon categorizing the patients in 3 groups according to age; <18 or ≥ 18 years and presence or absence of ETV as endoscopic procedure (see Table 4), we found in group 1, including adults without ETV, fever was present in 3/24 patients (12.5%). In group 2, including adults with ETV and children without ETV, fever was present in 25/125 (20%). In group 3, including children with ETV, fever was present in 31/58 (53%).

**Table 4:** Points distribution and patients’ groups

Points	0	1
Age	≥ 18 years (Adult)	<18 years (Child)
ETV performed	no	yes
**Group A = 0 points**	**Group B = 1 point**	**Group C = 2 points**
Adult without ETV	Adult with ETV / Child without ETV	Child with ETV

## Discussion

Postoperative fever is a common occurrence in pediatric neurosurgery, rarely indicating an underlying infection [2, 18]. The reported incidence of postoperative fever ranges from 35.6 to 61%. In the study by Goyal-Honavar et al., 35.6% of pediatric patients developed fever following neurosurgical procedures, with 16.4% of these cases linked to infection. Conversely, Raviv et al. found a higher incidence, with 61% of pediatric neurosurgical patients experiencing early postoperative fever, although only 1% of these cases were related to infection. More recent studies have shown a heightened association between neuroendoscopic procedures and postoperative fever, particularly in the first week after surgery [7, 18].

In our study, 61% of the 207 patients exhibited elevated body temperatures within the first postoperative week following neuroendoscopic procedures, with 28.5% developing fever (≥ 38.3 °C) at least once. Notably, there was a significant association between endoscopic third ventriculostomy (ETV) and the development of postoperative fever, with 71.2% of ETV patients experiencing elevated body temperature compared to 50.5% of those undergoing other neuroendoscopic procedures (*p* = 0.003). Similarly, postoperative fever (≥ 38.3 °C) was more frequent in the ETV group (37.5%) compared to the non-ETV group (19.5%, *p* = 0.005). However, there was no significant difference in subfebrile temperatures (37.5–38.2 °C) between the groups (33.7% in ETV vs. 31.1% in non-ETV, *p* = 0.767). These findings suggest that the manipulation of the third ventricular floor or hypothalamus during ETV and/or the structural changes and the possible irritation through CSF flow turbulence through ETV site likely contribute to postoperative temperature elevation. To avoid any bias of the results, all patients with proven infections were excluded from the study, and there were no significant differences between the two groups in terms of postoperative adverse events, clinical outcomes, or radiological improvements.

The ETV group exhibited a higher and more variable incidence of fever, peaking on Night 0 and fluctuating throughout the week, subsiding after Day 3. In contrast, the non-ETV group had a more stable and lower incidence of fever, with a peak on Day 1, followed by a gradual decline to 0% by Day 7.

Kinoshita et al. [9] were among the first to explore postoperative fever following neuroendoscopic procedures, reporting that 80.2% of their 86 cases developed fever (≥ 38.0 °C). In this study, the younger the patient, the more likely they were to develop postoperative fever, although the influence of ETV was not specifically addressed due to the small size of the comparison group. Similar to our findings, Kinoshita et al. observed that fevers often peaked on the day of surgery (45.3%) or on postoperative Day 1 (46.5%) and resolved spontaneously by Day 4 [9].

Focusing on the pediatric patients in our study, those who underwent ETV had a significantly higher tendency to develop postoperative subfebrile and febrile temperatures (> 37.49 °C) compared to those who did not undergo ETV (88% vs. 52%, *p* < 0.001). This pattern was also evident for fever (> 38.29 °C), with 53.5% of ETV patients developing fever compared to 21.5% of non-ETV patients (*p* < 0.001). Interestingly, adult patients were less likely to develop postoperative fever, supporting two hypotheses. First, while body temperature regulation is traditionally thought to be controlled by the anterior and median preoptic and dorsomedial hypothalamic nuclei [1, 4, 13], we hypothesize that manipulation of the third ventricular floor plays a role in this process. Second, the change in pressure and subsequent retraction of the lamina terminalis and floor of the third ventricle after ETV could also contribute to the development of fever. Pediatric patients, in particular, may exhibit greater flexibility in the lamina terminalis, making them more prone to postoperative temperature fluctuations following ETV [8]. Several studies have explored various aspects of ETV, examining its efficacy and success rates. These studies have also described anatomical changes in the third ventricular floor and the anterior curvature of the lamina terminalis as observed on MR imaging [5, 20, 21].

Kunder et al. also investigated postoperative fever in children undergoing neuroendoscopic procedures, finding that 70% of patients developed temperatures above 38.0 °C, with 22% experiencing fevers above 39.0 °C. Fever typically developed on the day of surgery or on the following day, with 25% of patients developing fevers on Day 0 and 49% on Day 1. Despite the high incidence of fever, only 13% of children were clinically ill, further supporting the notion that postoperative fever following neuroendoscopic procedures, particularly ETV, is rarely associated with infection [3].

After our risk stratification of occurrence of fever we concluded that presence of subfebrile temperature (37.5–38.2 °C) after endoscopic procedures is very common and not alarming of an infection. On the contrary, fever (> 38.2 °C) should be thoroughly investigated to rule out infection unless ETV was performed especially in children which explains up to 7 days of fever without presence infection of CSF.

### Limitations

The retrospective nature of this study must be acknowledged as a limitation, as it cannot reliably prove our hypothesis. The inhomogeneity of the study groups introduces an evident bias. Additionally, other factors contributing to the development of postoperative fever cannot be excluded and should be considered as potential biases, though they likely affect both study groups similarly. Further prospective and experimental studies are needed to explore the underlying mechanisms of postoperative body temperature elevation following endoscopic procedures, particularly after endoscopic third ventriculostomy (ETV).

## Conclusion

This study substantiates the hypothesis that manipulation of the floor of the third ventricle through endoscopic ventriculostomy may contribute to postoperative fever, rather than the neuroendoscopic procedure per se. Elevated temperatures were observed from the first night post-surgery and typically normalized by the third day without necessitating specific treatment. This phenomenon is particularly pronounced in pediatric patients following ETV. Further prospective studies are warranted to elucidate the precise mechanisms underlying intraoperative manipulation.

## Data Availability

No datasets were generated or analysed during the current study.
